# Sudden Death from Inflammation of the Peritoneum

**Published:** 1826-07

**Authors:** 


### SUDDEN AND MYSTERIOUS DEATH.

A young man (Lefevre) had been subject, during eighteen months, to pains in the epigastric region, which were aggravated by pressure—the tongue was occasionally white in the centre, and red at the sides—breath fœtid—bitter taste in the mouth—nausea—anorexia. To these symptoms were sometimes added diarrhœa, alternated with dryness of the skin and some febrile movement in the vascular system. These phenomena were generally dissipated by a few leeches, low diet, warm baths, and diluent drinks; and were always exasperated when the patient deviated from a low diet, and took stimulating food.

On the 2d November, 1824, Lefevre made a hearty breakfast, and was immediately afterwards seized with vomiting, restlessness, diarrhœa, and severe pains in the abdomen, coldness of the extremities, accompanied by feeble pulse and cold sweats. The symptoms were so extremely threatening, that M. Lombard, the medical attendant, conceived there was a perforation of the stomach or intestines, and prognosticated accordingly. Forty leeches were applied to the abdomen—with fomentations, &c. and M. Segelas was joined to the consultation. But no mitigation of the symptoms was produced, and Lefevre expired 17 hours after the commencement of the attack.

*Dissection.* On opening the abdomen a fetid gas was exhaled, and a considerable quantity of sero-sanguineous fluid escaped. The portion of peritoneum lining the abdominal parietes was covered with a multitude of small, round, and prominent tubercles, closely resembling an eruption of confluent small-pox. The intermediate spaces in the peritoneum were apparently healthy. The peritoneum reflected over the intestines was of a red rose colour, and presented a few of the said tubercles, as did the membrane where it is reflected over the mesentery and under surface of the diaphragm. There was no sign of perforation. There was a portion of small intestine, about a foot in length, of a livid red colour externally. There were some erosions in the mucous membrane of the stomach, which was softened and disorganized near the pyloric orifice. This was also the case in the mucous membrane of the duodenum. There were some erosions and marks of inflammation in the lining membrane of the small intestines, and upper portion of the colon.

This case shews how speedily mortal may sometimes be an acute inflammation supervening on a chronic.—*Archives*.

---

